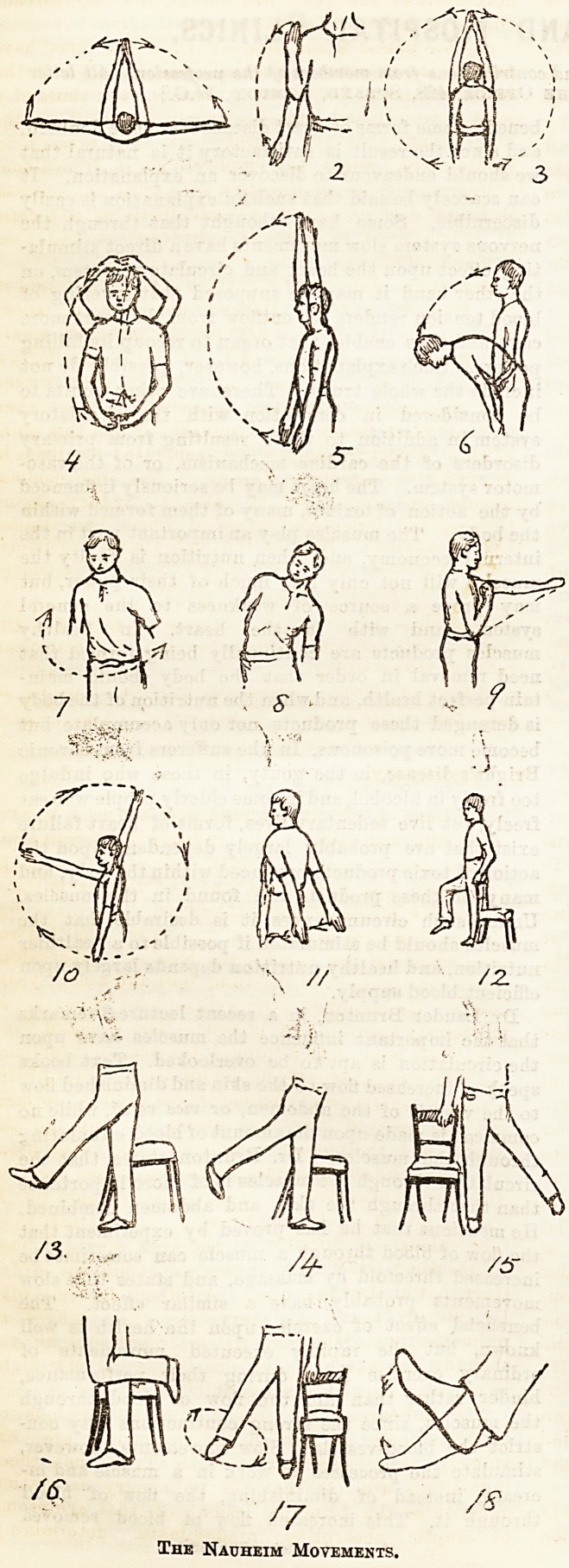# The Schott Treatment for Heart Disease

**Published:** 1896-01-11

**Authors:** Theodore Fisher

**Affiliations:** Pathologist to the Bristol Royal Infirmary.


### Medical Progress and Hospital Clinics.

[*The Editor will be glad to receive offers of co-operation and contributions from members of the profession. All letter should be addressed to* The Editor, at the Office, 428, Strand, London, W.C.]

### THE SCHOTT TREATMENT FOR HEART DISEASE.

By Theodore Fisher, M.D., Pathologist to the Bristol Royal Infirmary.

Dr. Greene has recently given in The Hospital a most lucid account of the Schott method of treatment for heart disease, but possibly the division of those methods dealing with the gymnastics or exercises cannot be clearly apprehended from a written description. It has been thought, therefore, that the few diagrams given below may be of some value to those readers who are interested in the subject. While attention is directed to these movements, it must not be supposed that they and they only are beneficial. But these movements have been designed with the object of bringing into play as many of the muscles of the body as possible, and, doubtless, answer the purpose as well as any. Other points have to be observed in addition to the direction of the movements. They are performed slowly, thirty to forty seconds being occupied by each, and slight resistance is offered by an attendant. This resistance is slight; if too great, it increases the tendency of the patient to hold his breath. This is to be avoided. The head should be held up, and the respiration be natural; any holding of the breath should be at once checked. Between every movement there is a pause of from thirty to forty seconds. The movements are continued for the space of about half-an-hour, and, although the same movement is never performed twice in succession, it may be necessary to repeat some of the earlier ones towards the end of the exercise.

1. Illustrates movement of the straightened arms horizontally across the front of the chest until the hands meet, and return of the arms to the starting point. 2. Illustrates flexion and extension of the forearm; movement of the opposite side follows. 3. The arms are raised perpendicularly above the head and lowered again. 4. The flexed index fingers are pressed against one another, and while they are kept in contact the arms are raised above the head. 5. The arms held parallel are raised forwards above the head and lowered again. 6. The trunk is flexed, then raised again to the upright position. 7. The trunk is rotated first to one side then to the other. 8. The trunk is flexed to one side; flexion on the opposite side follows. 9. Fist clenched, forearm flexed, and then straightened on arm to horizontal position. 10. Rotatory movement of arm; movement of opposite side follows. 11. Backward movement of both arms; arms after returned slowly to starting point. 12. Thigh flexed and returned to ordinary position. 13. Forward movement of leg and return. 14. Backward movement and return. 15. Outward movement and return. 16. Flexion of leg on thigh and return. 17. Rotatory movement of leg; movement of opposite side follows. 18. Flexion and extension of foot; movements of supination and pronation of the forearm are also performed, and flexion and extension of the wrist.

That the combined treatment by baths and exercises benefits some forms of heart disease cannot be doubted, and since the result is satisfactory it is natural that we should endeavour to discover an explanation. It can scarcely be said that such an explanation is easily discernible. Some have thought that through the nervous system slow movements have a direct stimulating effect upon the heart and circulatory system, on the other hand it may be supposed that lowering of blood tension renders the outflow from the heart more easy, and thus enables that organ to recoup its failing powers. Such explanations, however, possibly do not include the whole truth. There are other points to be considered in connection with the circulatory system in addition to those resulting from primary disorders of the cardiac mechanism, or of the vasomotor system. The heart may be seriously influenced by the action of toxines, many of them formed within the body. The muscles play an important part in the internal economy, and when nutrition is faulty the muscles will not only lose much of their power, but may prove a source of weakness to the general system, and with it the heart. In healthy muscles products are continually being formed that need removal in order that the body should maintain perfect health, and when the nutrition of the body is deranged these products not only accumulate but become more poisonous. In the sufferers from chronic Bright's disease, in the gouty, in those who indulge too freely in alcohol, and in some elderly people who eat freely, yet live sedentary lives, forms of heart failure exist that are probably largely dependent upon the action of toxic products produced within the body, and many of these products are found in the muscles. Under such circumstances it is desirable that the muscles should be stimulated if possible to a healthier nutrition, and healthy nutrition depends largely upon efficient blood supply.

Dr. Lauder Brunton, in a recent lecture,* remarks that the important influence the muscles have upon the circulation is apt to be overlooked. Text books speak of increased flow to the skin and diminished flow to the vessels of the abdomen, or *vice versâ*, while no comment is made upon the amount of blood circulating through the muscles. Dr. Brunton states that the circulation through the muscles is of more importance than that through the skin and abdomen combined. He mentions that he has proved by experiment that the flow of blood through a muscle can sometimes be increased threefold by massage, and states that slow movements probably have a similar effect. The beneficial effect of exercise upon the health is well known, but the rapidly executed movements of ordinary exercise may, during their performance, hinder rather than aid the flow of blood through the muscles, since the strong contractions may constrict the blood-vessels. Slow movements, however, stimulate the processes at work in a muscle and increase, instead of diminishing, the flow of blood through it. This increased flow of blood removes

* *Lancet*, October 12th, 1895.

THE NAUHEIM MOVEMENTS.

harmful products of metabolism and stimulates to healthier nutrition. The health of the muscles improves, and with them the whole body, including the heart. If such a theory be correct, cases of heart failure in which valvular disease is absent ought to improve most markedly, and examples of such failure are undoubtedly most benefited. Cases of valvular disease, however, also improve, and in such disease there is a similar failure of nutrition of the various organs of the body, including the muscles, this failure being the consequence rather than the cause of the heart failure. Improvement of nutrition, however, reacts beneficially upon the heart, and no doubt delays the final breakdown.

In closing, it may be said that we do not intend to imply that the whole system known as the Schott treatment consists of improved nutrition of the muscles; influences appear to be at work that are difficult to understand, at least in connection with the baths. For example, a patient at Nauheim, a commonsense man of business, told me that on commencing treatment he was unable to walk from his hotel to the bath houses without a rest on the way, but that after his bath he could easily walk back. The immediate effect shown in some cases seems obscure, but that such effects are produced prove that the system is a valuable aid in the treatment of heart disease. One feels, however, that improvement must not be expected in every case. If expectations are raised too high there may be disappointment; but if the results of the methods be examined without prejudice, we shall learn that they may be applied with benefit to patients and with credit to the profession.

## Figures and Tables

**2 3 4 5 6 7 8 9 10 11 12 13 14 15 16 17 18 f1:**